# Antioxidant Activity of Essential Oil Extracted by SC-CO_2_ from Seeds of *Trachyspermum ammi*

**DOI:** 10.3390/medicines4030053

**Published:** 2017-07-11

**Authors:** Aarti Singh, Anees Ahmad

**Affiliations:** Department of Chemistry, Aligarh Muslim University, Aligarh 202002, UP, India; aarthi.chem2013@gmail.com

**Keywords:** SC-CO_2_, sustainable, optimization, GC-MS, FTIR, HPTLC

## Abstract

**Bcakground:** Extracts obtained from natural sources such as plants are of immense importance for humans. **Methods:** Therefore this study was conducted to obtain essential oil from the seeds of *T. ammi* by conventional and non-conventional methods. Hydrodistillation (HD), Solvent Extraction (SE), Ultrasonication (US), and Supercritical Carbon-dioxide (SC-CO_2_) extraction techniques were used to extract essential oil from the powdered seeds of *T. ammi*. A quality control method for each extracted oil was developed using HPTLC, FTIR, and GC-MS. The optimization process was carried out using fractional factorial design (FFD) under which three parameters were considered: pressure (150, 175, and 300 bar), temperature (25, 30, and 40 °C), and CO_2_ flow rate (5, 10, 15 g/min). **Results:** The yield of essential oil obtained from the HD, SE, US, and SC-CO_2_ methods were 1.20%, 1.82%, 2.30%, and 2.64% *v/w*, respectively. Antioxidant activity was determined by the DPPH and superoxide scavenging methods and the *IC*_50_ (Inhibition Concentration) values of the *T. ammi* oil sample were found to be 36.41 and 20.55 µg mL^−1^, respectively. **Conclusion:** The present paper reported that different extraction methods lead to different yields of essential oils and the choice of a suitable method is extremely important to obtain more preferred compounds. The yield was higher in the SC-CO_2_ method and it is a sustainable and green extraction technique. Many important constituents were detected in analytical techniques. Antioxidant activities carried out showed that essential oil extracted from *T. ammi* seeds possess significant antioxidant activity.

## 1. Introduction

*Trachyspermum ammi* L. is an annual herb which stands erect at 30 to 90 cm with small white flowers and belongs to the family Apiaceae. It is also known by other common names such as, Sanskrit: *Yamini, Yaminiki, Yaviniki*, Assamese: *Jain*, Bengali: *Yamani, Yauvan, Yavan, Javan, Yavani, Yoyana*, English: *Bishop’s weed*, Gujrati: *Ajma, Ajmo, Yavan, Javain*, Hindi: *Ajwain, Jevain*, Kannada: *Oma, Yom, Omu*, Malayalam: *Oman, Ayanodakan*, Marathi: *Onva*, Oriya: *Juani*, Tamil: *Omam*, Telugu: *Vamu*, Urdu: اجوائن‎‎ajwan [1]. It is known to be native to Egypt and is also cultivated in Iraq, Iran, Afghanistan, Pakistan, and India [1]. Gujarat and Rajasthan are its major cultivating regions in India. Thymol (2-isopropyl-5-methylphenol) is a natural monoterpene phenol derivative of cymene, C_10_H_14_O, isomeric with carvacrol. It has been reported in many plants which contain thymol as major component, such as *Trachyspermum ammi* (Ajwain), *Monarda didyma*, *Monarda fistulosa*, *Origanum dictamnus*, *Origanum compactum*, *Origanum dictamnus*, *Origanum onites*, *Origanum vulgare*, *Thymusglandulosus*, *Thymus hyemalis*, *Thymus vulgaris*, *Thymus zygis*, and *Satureja hortensis* etc. [2,3,4,5]. *Trachyspermum ammi* (L) Sprague is a Greek word Trachy that means rough and spermum means seeded, whereas ammi is name of the plant in Latin Syn. *Carum copticum*, commonly known as Joan belonging to the family Apiaceae or Umbelliferae [6]. It has been found to be a medicinally valued seed and has shown various pharmacological activities like antioxidant, antinociceptive, cytotoxic, antiviral [7], anti-inflammatory [8], antifungal [9,10,11,12,13], molluscicidal [14,15,16], antihelminthic (in sheep) [17,18], plant nematicidal [17,19], antipyretic [20], antiaggregatory [21] and antimicrobial activity [22,23,24], hypolipidemic [25], broncho-dilating actions, antilithiasis, diuretic, antitussive [26], abortifacient, antihypertensive, antifilarial [27], and antispasmodic activities.

In this study, essential oils from the seeds of *T. ammi* were extracted using conventional and non-conventional methods (HD, SE, US, and SC-CO_2_) to compare the yield, the SC-CO_2_ extraction process was optimized by using FFD, the composition of the essential oil was determined by the use of HPTLC, GC-MS, and FTIR, and the antioxidant activity was determined by the DPPH and superoxide scavenging methods.

## 2. Materials and Methods

### 2.1. Plant Materials

The plant material (seeds of *T. ammi*) was collected from a local market in Aligarh. Dried *T. ammi* seeds were ground in a mechanical grinder for a short but sufficient period of time (30 s) to obtain a uniform particle size (0.01–3500 mm) distribution. The grounded powder was sieved.

### 2.2. Chemicals

Every single ingredient taken was of Pharmacopeial quality and quantity. All the chemicals and standards were procured from Sami Labs Ltd. (Bangalore, India). Ethanol, ether, hydrochloric acid (HCL), potassium hydroxide (KOH), ethanolic potassium hydroxide solution (60%), ethanolic hydroxylamine hydrochloride solution (90%), 10% Folin-Ciocalteu, Na_2_CO_3_ (Sodium carbonate), methanol, standard (Gallic acid), DPPH (Diphenyl-1-picrylhydrazyl), phenazine methosulfate (PMS), standard (Ascorbic acid), nitroblue tetrazolium (NBT), and nicotinamide adenine dinucleotide (NADH) were obtained from Merck Ltd., (Bangalore, India) and were used. Carbon dioxide was obtained from Sigma gases (Gupta Gases, New Delhi, India).

### 2.3. Experimental Design

#### 2.3.1. Full Factorial Design (FFD)

FFD is an experiment whose design consists of two or more factors, each with discrete possible values or “levels” and whose experimental units take on all possible combinations of these levels across all such factors. When conducting an experiment, varying the levels of all of the factors at the same time instead of one at a time allows us to study the interactions between the factors. In FFD, responses are considered at all combinations of experimental factor levels [28]. Each experimental condition is called a “run” and each run represents a variation of one variable. Each response will be measured for an observation. The entire set of runs is the “design”. Since we consider three parameters with three levels, the total run with a full factorial design is 3^3^ (27) number of trials. The various levels for the parameters are shown in Table 1.

### 2.4. Extraction Methods

#### 2.4.1. Hydrodistillation Method

In hydrodistillation, the powdered plant material is mixed with distilled water and heated to release the essential oil. The seeds of *T. ammi* were dried and ground. The grounded seed powder (150 g) and distilled water (500 mL) was mixed in a 1 L round bottom flask and then was heated for 5 h to extract the essential oil [29]. In order to separate small water droplets present in the extracted essential oil, the extract was centrifuged at 10,000 rpm for 10 min. The centrifuged oil was kept at 4 °C until further analysis and the percent yield was calculated.

#### 2.4.2. Solvent Extraction

In solvent extraction, the powdered plant material is mixed with organic solvent which extracts the essential oil. The coarse powder (50 g) was extracted with hexane (100 mL) in a 1:2 *w/v* ratio, and was then kept in a conical flask for 24 h. The extract was filtered and concentrated under reduced pressure in a rotary vacuum evaporator at 40 °C. The concentrated oil was stored at low temperature (4 °C) prior to analysis and the percent yield was calculated.

#### 2.4.3. Ultrasonic Assisted Extraction

In ultrasonic assisted extraction, moderate temperature is used, the solvent amount is reduced, and the extraction time is shortened. Fifty grams of coarse powdered plant material was taken in a conical flask (250 mL) and 100 mL of hexane (1:2 *w/v*) was added to it. The flask was covered and then placed in an ultrasonic water bath (at 25 °C) apparatus for 30 min (frequency 33 kHz). After that, the extract was filtered and concentrated in a rotary vacuum evaporator (Atico Medical Pvt. Ltd., Ambala, India) under reduced pressure. The concentrated oil was then stored at low temperature (4 °C) prior to analysis and its percent yield was calculated.

#### 2.4.4. Supercritical CO_2_ Extraction

SC-CO_2_ extraction is a green and environmentally friendly technique in which no harmful organic solvent is used, the extraction is carried out at low temperature that is useful for the extraction of thermolabile compounds, and the extract obtained is pure. The extraction vessel was made of stainless steel with the capacity of 200 mL. Ground powder of the plant material (50 g) was filled in the extractor and then CO_2_ was allowed to flow through the powdered plant material at the required pressure (150–300 bar), temperature (25–40 °C), and flow rate (5–15 g min^−1^) [30]. The total time for extraction of the essential oil was 30 min. The extracted essential oil was then present in the collecting vessel from where it is collected into vials [31,32]. The essential oil was stored at low temperature around 4 °C until further analysis and its percent yield was calculated.

### 2.5. Analytical Methods

#### 2.5.1. Scanning Electron Microscopy (SEM) Analysis

SEM produces images of a sample by scanning the surface with a focused beam of electrons. The electrons interact with the atoms at various depths within the sample which produces various signals that contain information about the sample’s surface topography and composition. A SEM Model JSM-6510LV, JEOL (Tokyo, Japan) was used to study the surface morphology of the oily and non-oily biomass of the *T. ammi* seeds before and after SC-CO_2_ extraction, respectively. The SEM was operated at 10 kV, with a working distance of 11 and 12 mm, at magnifications of 1000× and 2000×.

#### 2.5.2. High Performance Liquid Chromatography (HPTLC)

The samples were spotted in the form of a band (3.0 mm) with a Camag microlitre syringe on a TLC aluminium plate precoated with silica gel 60F-254 (20 × 10 cm with 0.2 mm thickness, E. Merck, Berlin, Germany) using a Camag Linomat V sample applicator. A constant application rate of 120 nL s^−1^ was employed and the space between two bands was 8.0 mm. The slit dimension was kept at 3.0 × 0.20 mm and a scanning speed of 20 mm s^−1^ was employed. The development was carried out in a linear ascending manner in a twin trough glass chamber (20 × 10 cm) saturated with the mobile phase composed of hexane:ethyl acetate:formic acid in a ratio of 7:2:1 *v/v/v*. The optimized chamber saturation time for the mobile phase was 20 min at room temperature and the chromatogram was developed up to the length of 85 mm. The TLC plate was then dried. The HPTLC plates were observed and studied specifically at 254 nm and 366 nm as well as in the visible range (580 nm) after spraying with anisaldehyde sulfuric acid reagent. A good separation of constituents was observed at 580 nm.

#### 2.5.3. Gas Chromatography-Mass Spectrometry (GC-MS)

GC is used for separating and analyzing compounds that can be vaporized without decomposition. The essential oil obtained by the different extraction techniques was analysed using GC (Agilent Technologies, Santa Clara, CA, USA) interfaced with a MS ion-trap detector. The separation was obtained using a 5% phenyl polymethyl siloxane capillary column (Agilent Technologies, USA-Germany) (30 m × 0.25 mm i.d., thickness 0.25 µm). Helium was used as the carrier gas (1 mL/min). The carrier gas temperature was kept at 65 °C. The injector was maintained at 65 °C. The column temperature was raised at a rate of 10 °C/min from 160 °C to 302 °C. Splitless sample injection (2 µL) was used. The ionization energy for the mass spectrometer was 70 eV. The essential oils obtained by the different extraction techniques were diluted by adding 1998 µL of hexane to 2 µL oil (Hydro-distilled oil) and 1990 µL hexane to 10 µL oil (extracted by other techniques) under optimized operating conditions.

By comparing with the National Institute of Standard and Technology (NIST) library, the compounds were detected and identified [33].

#### 2.5.4. Fourier Transform Infrared Spectroscopy (FTIR) Spectral Analysis

Fourier transform infrared (FTIR) spectral analysis was performed by using a Shimadzu BioRad FTIR (Kyoto, Japan). The samples were dispersed and triturated with dry potassium bromide (2 µL of the sample). It was then finely ground using a mortar and pestle to prepare the KBr disk at a pressure of 1000 psig. The prepared disk was placed in the FTIR sample holder, where the spectra in absorbance mode in the spectral region from 4000 to 400 cm^−1^ was obtained using the resolution of 4 cm^−1^.

## 3. Antioxidant Activity

### 3.1. Diphenyl-1-Picrylhydrazyl (DPPH) Free Radical Scavenging Method

In this method, the stock solutions (1 mg/mL) were prepared by mixing samples with 95% methanol. One hundred µL of 0.5 mM 2,2-diphenyl-1-picrylhydrazyl radical (DPPH) in methanol was mixed with 100 µL of the samples in 96 well plates at various concentrations (0.781, 1.56, 3.12, 6.25, 12.5, 25.0, 50.0, and 100.0 µg) in duplicate. The 96 well plates were allowed to stand at room temperature for 30 min in dark conditions. The control was prepared as described above without the sample or standards, whereas the blank was prepared without DPPH containing sample and methanol. The changes in absorbance of all of the samples and standards were measured at 540 nm in an Elisa plate reader (Bio Rad 680, PerkinElmer, Bangalore, India). The radical scavenging activity was calculated using the corrected ODs (COD) of the control and samples as per Equations (1) and (2).

COD control = OD control − OD control blank
(1)

(2) $$\mathrm{Radical}\;\mathrm{scavenging}\;\mathrm{activity}\;\left(\%\right)=\frac{\mathrm{COD}\;\mathrm{control}\;–\;\mathrm{COD}\;\mathrm{sample}}{\mathrm{COD}\;\mathrm{control}\;\times \;100}$$

*IC*_50_, which is the concentration of the sample required to scavenge 50% of the free radicals, was calculated [34].

### 3.2. Super Oxide Anionic Scavenging Method

Various concentrations ranging from 10 to 50 μg/mL of the sample (0.3 mL) were taken. The superoxide anion was produced in 3 mL of phosphate buffer (100 mM, pH 7.4) containing 0.75 mL of NBT (300 μM) solution and 0.75 mL of NADH (936 μM) solution. The reaction was initiated by the addition of 0.75 mL of PMS (120 μM) to the mixture. The absorbance at 560 nm (Schimadzu UV-Vis 1601, PerkinElmer, NJ, USA) was measured in a spectrophotometer after incubation at room temperature for a duration of 5 min. The super oxide anion scavenging activity was calculated according to Equation (3).

% Inhibition = [(A_0_ − A_1_)/A_0_ × 100] (3)

where A_0_ is the absorbance of the control (without extract) and A_1_ is the absorbance of the extract or standard.

The concentration of the sample required to scavenge 50% of the free radicals, known as *IC*_50_, was calculated [35].

### 3.3. Statistical Analysis

The data obtained from the SC-CO_2_ extraction (pressure, temperature, flow rate of CO_2_) were subjected to analysis of variance (ANOVA) to determine the significant difference among all the extract yields. A *p*-value less than 0.05 was considered significant. The MINITAB 14 statistical software (version of OMNITAB, Minitab Inc., Pennsylvania State University) package was used to perform all statistical analyses.

## 4. Results and Discussion

### 4.1. Percentage Yield (% v/w)

Different extraction techniques (HD, SE, US, and SC-CO_2_) were carried out in order to obtain the maximum yield. Oil obtained by SC-CO_2_ (2.64%) had the maximum yield, whereas 1.20%, 1.82%, and 2.30% were obtained from the HD, SE, and US methods, respectively. Most of the compounds were degraded in HD due to the high temperature. In the case of SE, harmful organic solvents are used. SC-CO_2_ is the best extraction technique in order to obtain a high yield of the extract qualitatively and quantitatively.

### 4.2. SEM Analysis

**Figure 1:**
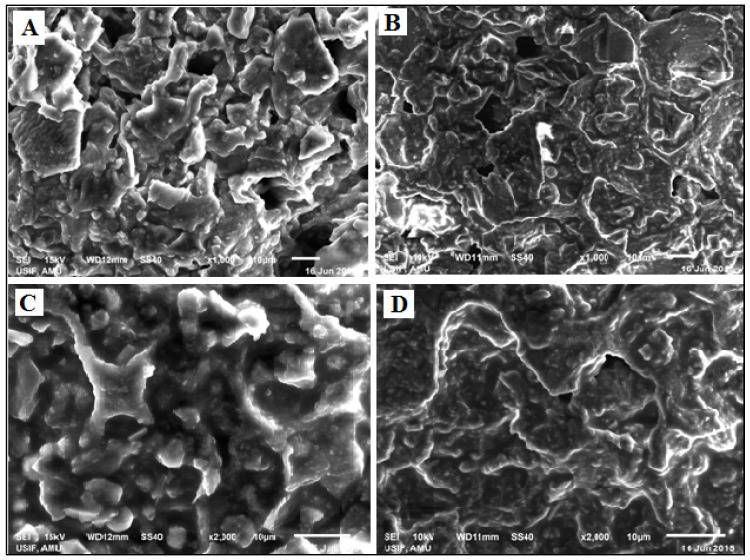
SEM images of *T. ammi* L. seed biomass: (**A**) before SC-CO_2_, (**B**) after SC-CO_2_ at 1000× magnification, and (**C**) before SC-CO_2_, (**D**) after SC-CO_2_ at 2000× magnification.

SEM images of the oily and non-oily biomass were captured before and after SC-CO_2_ extraction at 1000× and 2000× magnification. Figure 1 shows that before SC-CO_2_ extraction, the oily biomass of the seeds of *T. ammi* has smoother surfaces of the oil glands filled with oil. After SC-CO_2_ extraction, the non-oily biomass appears smashed with rugged features and rough edges. Thus the images show that most of the oily fraction has been extracted, which is in agreement with the high yield obtained with SC-CO_2_. CO_2_ as a gas was not retained by the matrix and thus SEM offers more depth profile information than conventional light microscopy to understand the sample morphology.

### 4.3. Solvent System for TLC

The constituents of the essential oil extracted by the various techniques were separated using TLC. Various solvent systems in different ratios were used for the separation of the phytoconstituents present in the essential oils. The list of various solvent systems used is provided in Table 2. The solvent system containing hexane:ethyl acetate:formic acid (7:2:1, *v/v/v*) gave the maximum separation of constituents present in the essential oil and compactness of bands for all the essential oil samples.

### 4.4. Detection of Spots of Different Samples

**Figure 2:**
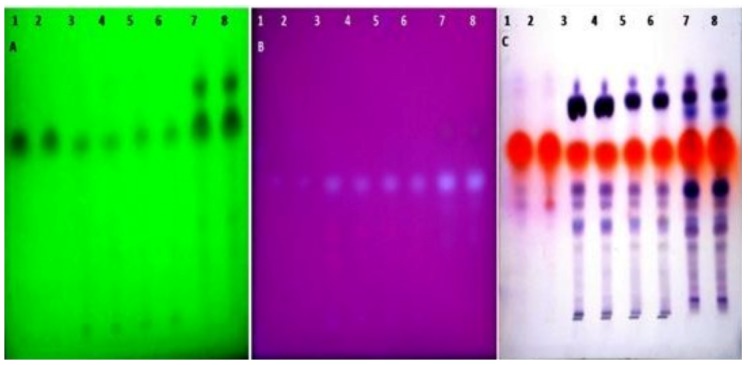
HPTLC fingerprint of different essential oils of seeds of *T. ammi* L. extracted using different extraction techniques (track 1–2: Hydrodistilled oil, 3–4: Solvent extracted oil, 5–6: Ultra sonication oil, 7–8: SC-CO_2_ oil) observed at (**A**) 254 nm, (**B**) 366 nm, (**C**) In daylight after derivatization by using the anisaldehyde sulphuric acid reagent.

The HPTLC plates were prepared, observed, and studied specifically at 254 nm, 366 nm, and at 580 nm in the visible range after spraying with the anisaldehyde sulfuric acid reagent, as shown in Figure 2. It was found that a good separation of constituents was observed at 580 nm.

**Figure 3:**
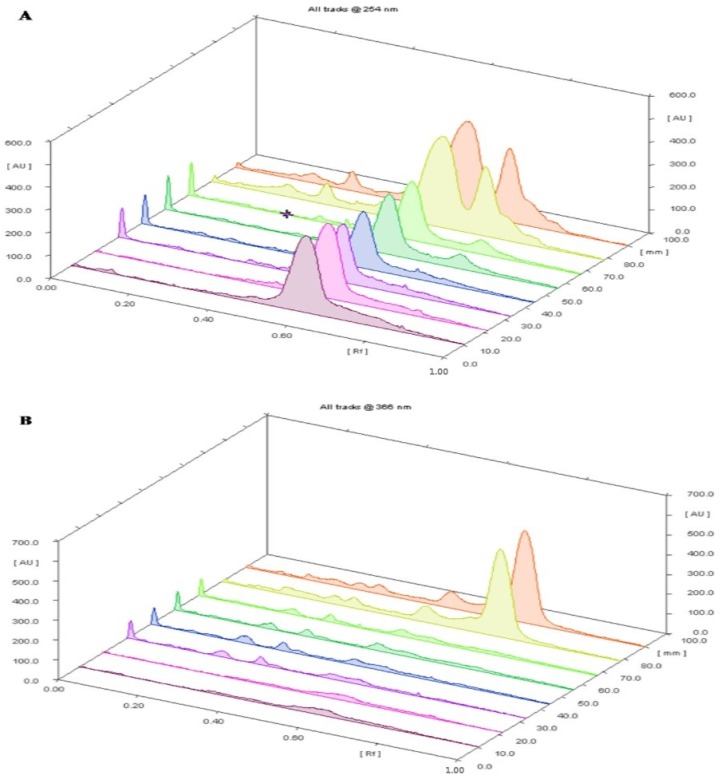

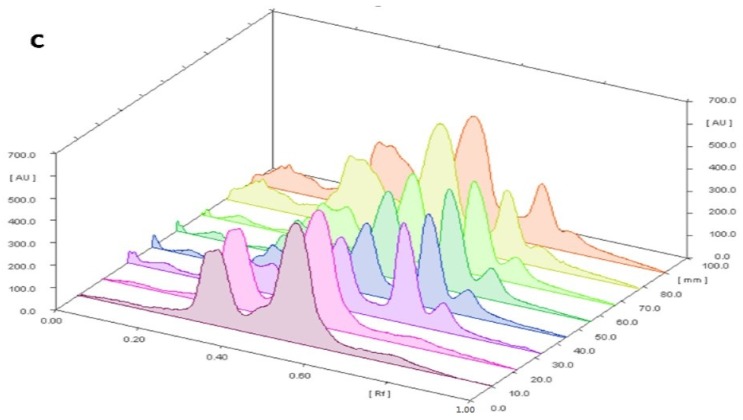
(**A**) 3D Chromatogram of 8 tracks of *T. ammi* L. oil obtained by different extraction techniques at A: 254 nm; (**B**) 3D Chromatogram of 8 tracks of *T. ammi* L. oil obtained by different extraction techniques at B: 366 nm; (**C**) 3D Chromatogram of 8 tracks of *T. ammi* L. oil obtained by different extraction techniques at B: 580 nm.

**Figure 4:**
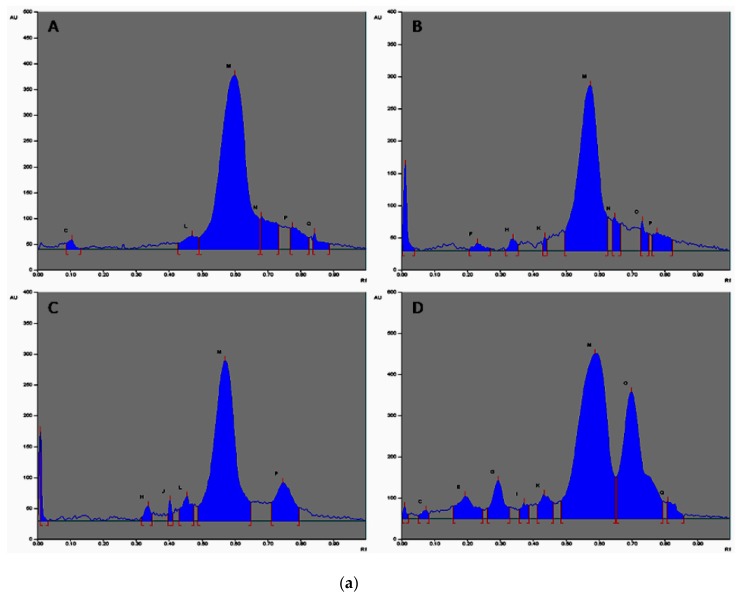

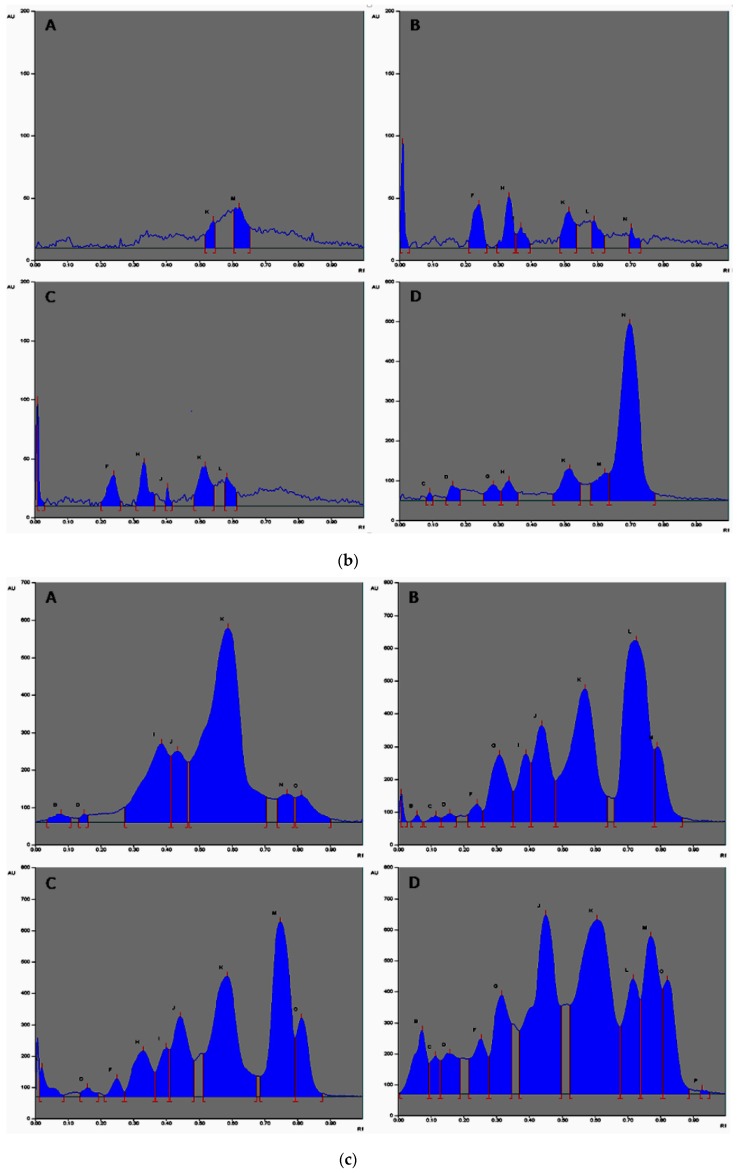
(**a**) HPTLC fingerprint of various essential oils of seeds of *T. ammi* L. extracted via diverse extraction methods. A: Hydrodistilled oil, B: Solvent extracted oil, C: Ultra sonicated oil, D: SC-CO_2_ oil observed at 254 nm with assigned substance; (**b**) HPTLC fingerprint of various essential oils of seeds of *T. ammi* L. extracted using different extraction methods. A: Hydrodistilled oil, B: Solvent extracted oil, C: Ultra sonicated oil, D: SC-CO_2_ oil observed at 366 nm with assigned substance; (**c**) HPTLC chromatograms of different oils of *T. ammi* L. Extracted using different extraction techniques. A: Hydrodistilled oil, B: Solvent extracted oil, C: Ultra sonication oil, D: SC-CO_2_ oil visualized at 580 nm with assigned substance.

The comparative results of the different oils showed a dominant constituent at the R_f_ value 0.58 at 254 nm in the oil extracted by different extraction techniques at different area percentages. Substance N at R_f_ 0.66 showed a higher area percentage in the oil extracted by the supercritical critical fluid extraction technique at 366 nm as compared to the other extraction techniques. Substance M was present in maximum amounts in all of the oil samples at 254 nm and in only solvent extracted and supercritical fluid extracted oil at 366 nm. Substances A, E, G, and I were present in only the supercritical fluid extracted oil at 254 nm. 3D Chromatograms and HPTLC fingerprinting of the essential oils obtained from the various extraction techniques scanned at 254 and 366 nm are shown in Figure 3A,B, Figure 4a,b, respectively. The comparative results of the different oils are given in Table 3.

The HPTLC plate scanned at 580 nm (Figure 3c and Figure 4c) showed the maximum area percentage at Rf (0.59) in the oil extracted by the hydrodistillation process. Substances K and J are present with the maximum area percentage in all of the extracted techniques, as shown in Table 4.

### 4.5. GC-MS Analysis

**Figure 5:**
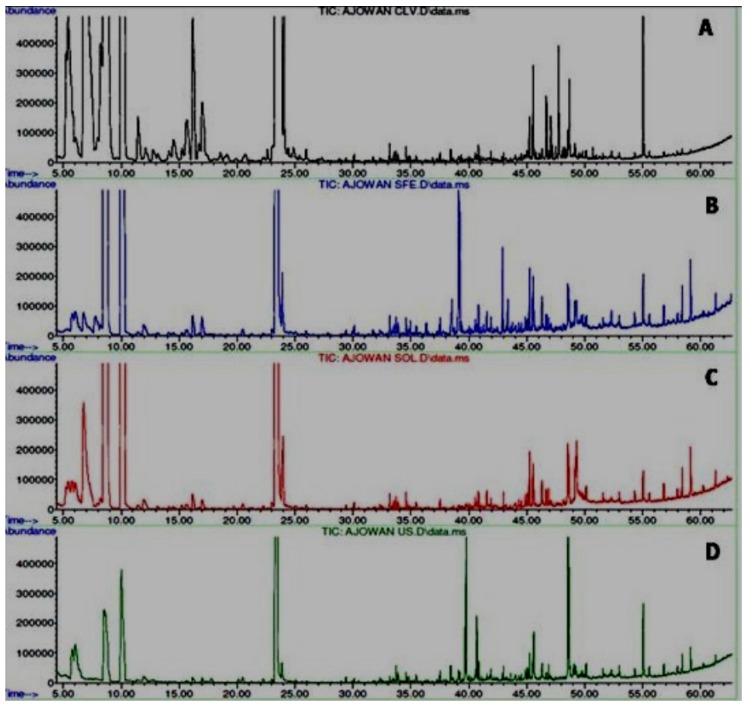
Comparative GC-MS chromatograms of different oils of *T. ammi* L. extracted using different extraction techniques. (**A**) Hydrodistilled oil, (**B**) Solvent extracted oil, (**C**) Ultrasonicated oil, (**D**) SC-CO_2_ oil.

GC-MS chromatograms of the essential oils of seeds of *T. ammi* L. extracted by various methods, including conventional (HD, SE, US) and non-conventional (SC-CO_2_) techniques, are shown in Figure 5. Around 49 compounds were identified in the chromatographic analysis (GC/MS) of the essential oils using their retention indices and mass spectra fragmentation from the Nist and Wiley library, as well as by comparison of the fragmentation patterns of the mass spectra with those reported in the literature data [36] (Table 5). Only 26 components were identified [37] using the solvent extraction method and only 7 components were identified in the *T. ammi* oil extracted by the hydrodistillation method [38,39].

Thymol (69.25%) and γ-terpinene (23.62%) were the main constituents of all of the samples. Other components present were eudesmol (1.28%) and 3-Thujene (1.20%). Fatty acids were also present in significant quantities.

### 4.6. FTIR Analysis

**Figure 6:**
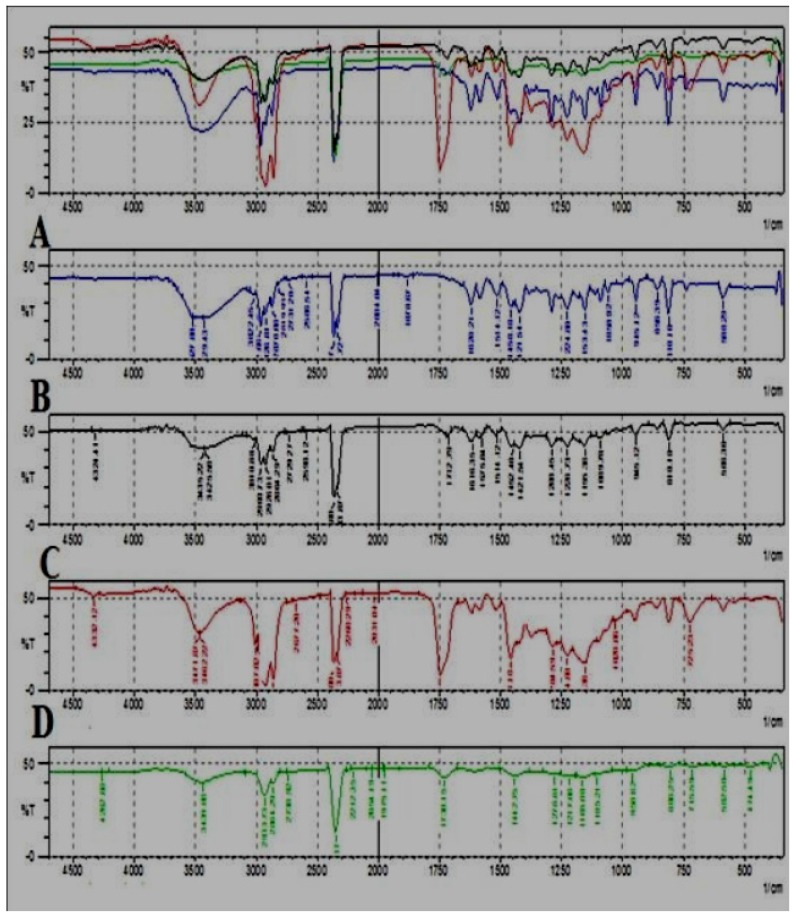
Comparative FTIR spectra of different oils of *T. ammi* L. extracted using different extraction techniques. (**A**) Hydrodistilled oil, (**B**) Solvent extracted oil, (**C**) SC-CO_2_ oil, (**D**) Ultrasonicated oil.

The elucidation of the infrared spectra of the essential oils obtained from the seeds of *T. ammi* was carried out by comparing the absorption bands in the spectrum with the known absorption frequency bands. The FTIR spectra of the essential oils extracted by various extraction methods are shown in Figure 6, and the results are shown in Table 6.

The IR spectrum for *T. ammi* oil exhibits a medium intensity band at 3429 cm^−1^ assigned to the stretching vibration of the amine group. The peak at 2962–2729 cm^−1^ is due to the stretching of the CH_3_ group, and the peak at 1458 cm^−1^ represents C=C aromatic ring stretching. The peak at 810 cm^−1^ is due to out-of-plane alkene C-H stretching. The C-H stretching medium intensity peak of the aldehydic group is present in the HD, SE, and SFE oil samples. A band appearing at 1155.36 cm^−1^ represents the strong stretching vibration of the alcoholic group present in all samples except for the ultrasonicated oil sample.

### 4.7. Antioxidant Activity

**Figure 7:**
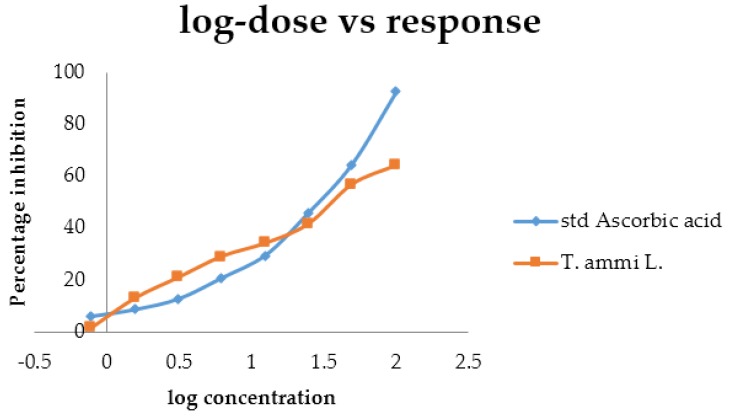
Comparative dose response curve between percent inhibitions against log concentration by the DPPH method.

The *T. ammi* oil sample showed a significant concentration-dependent antioxidant activity by inhibiting the DPPH free radical with an IC_50_ value of 36.41 μg mL^−1^, whereas, an *IC*_50_ value for ascorbic acid was found to be 28.09 μg mL^−1^ used as a standard (Table 7 and Figure 7). It was found that the oil possesses hydrogen donating capabilities that are similar to ascorbic acid and acted as an antioxidant. The scavenging effect increased with increasing concentration of the extract and ascorbic acid (5.0–100 μg mL^−1^).

**Figure 8:**
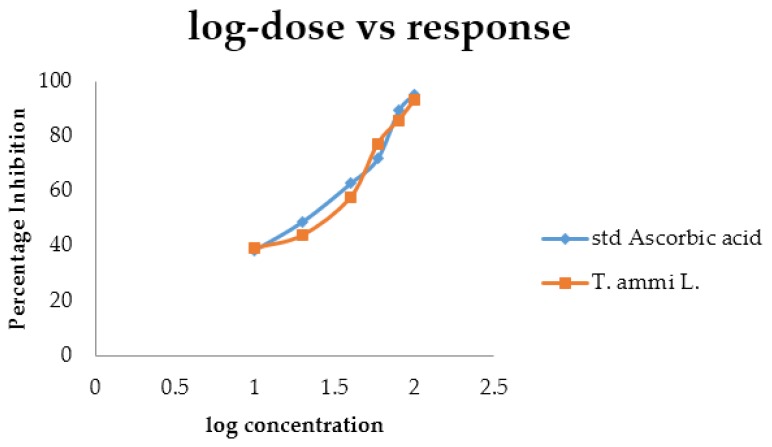
Comparative dose response curve between percent inhibitions against log concentration by the superoxide scavenging method.

In the case of the superoxide scavenging method, the activity of the drug increased noticeably with the increase in concentration. Comparative data of the antioxidant activity of the extracts and ascorbic acid are shown in Table 8 and Figure 8. The *IC*_50_ values of ascorbic acid and the essential oil were found to be 20.14 μg mL^−1^ and 20.55 µg mL^−1^, respectively.

### 4.8. Full Factorial Design (FFD)

**Figure 9:**
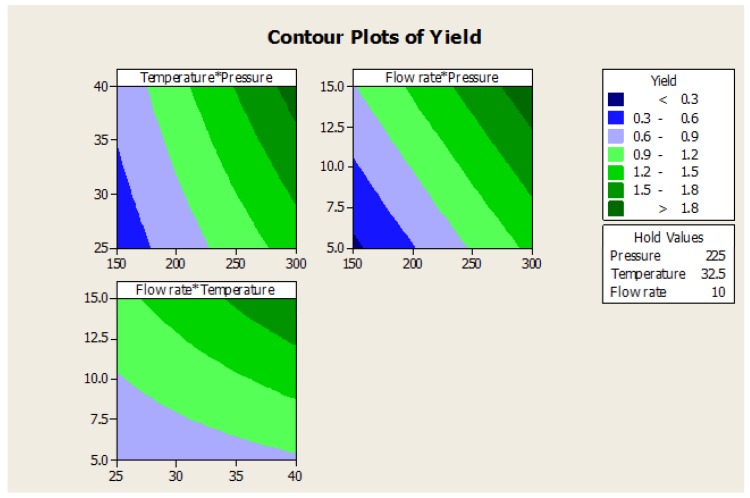
Contour plots of yield vs. pressure, temperature, and flow rate.

**Figure 10:**
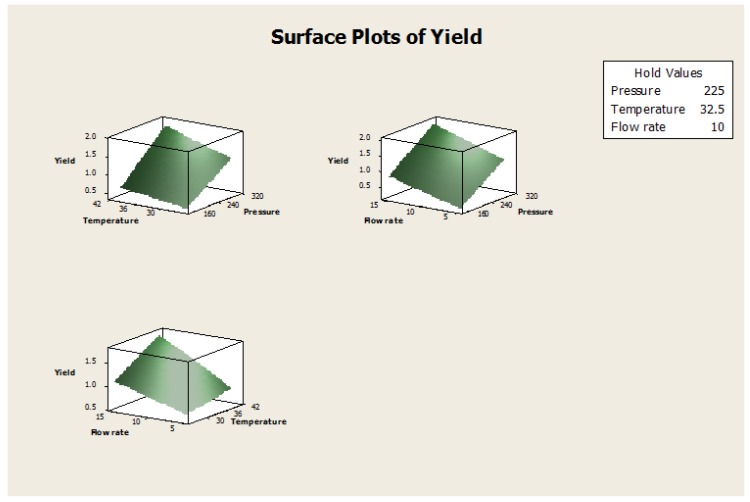
Surface plots of yield vs. pressure, temperature, and flow rate.

The FFD experiment was carried out to determine the effect that three factors (pressure, temperature, flow rate) with three levels had on the response (Yield). The results were analyzed and are shown in Table 9. It can be seen that the selected factors have a significant effect either directly or indirectly through the interaction with other factors. The selected factors were 85.42% fit model for the given data. The contour and surface plots have been shown in Figure 9 and Figure 10.

The three factors of pressure, temperature, and flow rate were shown to have direct effects on the yield with *p* < 0.05, hence the pressure, temperature, and flow rate are significant factors that influence the yield. While a combination of temperature-flow rate affects the yield to a certain extent as *p* < 0.05, on the other hand, pressure-flow rate, pressure-temperature, and pressure-temperatute-flow rate combinations have no significant effect on the oil yield.

The optimum operating conditions for SC-CO_2_ extraction of the essential oils from the seeds of *T. ammi* with high yield were: pressure (225 bar), temperature (32.5 °C), and flow rate of CO_2_ (10 g/min). A confirmation experiment was run using the optimum conditions and the highest yield obtained was 2.64%.

## 5. Conclusions

In the present work, the essential oil from seeds of *T. ammi* was extracted via HD, SE, US, and SC-CO_2_ Extraction methods. Out of all of these methods, SC-CO_2_ produced the highest percentage yield (2.64%) as compared to the other extraction techniques. FFD with the optimum conditions of pressure (225 bar), temperature (32.5 °C), and flow rate of CO_2_ (10 g/min) produced the maximum yield. The statistical analysis shows that pressure, temperature, and flow rate affect the yield of the oil and the combination of temperature-flow rate also shows significant influence on the yield with *p* < 0.05, while pressure-flow rate, pressure-temperature, and pressure-temperature and flow rate combinations have no effect on the oil yield with *p* > 0.05. In addition, the results of the HPTLC, GC-MS and FTIR analyses have shown that the SC-CO_2_ extraction technique is the best technique for the extraction of essential oils. SC-CO_2_ is a green, environmental friendly, and sustainable extraction technique. The antioxidant activity, which was determined by the DPPH and superoxide anion scavenging methods, was found to be 36.41 and 20.55 µg mL^−1^, respectively. Hence it was concluded that the essential oil extracted from the seeds of *T. ammi* possess significant antioxidant activity.

## Figures and Tables

**Table 1:** Parameters and levels used in the experimental design.

Level	Factors		
	Pressure (bar)	Temperature (°C)	CO_2_ Flow Rate (g/min)
1	150	25	5
2	175	30	10
3	300	40	15

**Table 2:** Solvent system tried for separation of the phytoconstituents of essential oil.

Serial No	Solvent System	Ratio (*v/v/v*)	Observation
1	Hexane:dichloromethane	95:5	No separation
2	Toulene:diisopropyl ether:ethyl acetate	80:10:10	No separation
3	Toulene:diisopropyl ether	95:5	No separation
4	Petroleum ether:ethylacetate:formic acid	95:5:1	No separation
5	Cyclohexane:ethylacetate	90:10	No separation
6	Chloroform:methanol:formic acid	90:2:1	Little separation
7	Chloroform:methanol:formic acid	90:3:1	Little separation
8	Chloroform:methanol:formic acid	90:1:1	Little separation
9	Toulene:methanol:formic acid	90:7.5:1	Little separation
10	Toulene:methanol:formic acid	90:6:1	Little separation
11	Toulene:chloroform:formic acid	90:2:1	Little separation
12	Toulene:acetone	95:5	No separation
13	Toulene:ethyl acetate	97:5	Fair
14	Toulene:ethyl acetate	97:3	Fair
15	Toulene:ethyl acetate:formic acid	95:5:1	Fair
16	Hexane:ethyl acetate:formic acid	90:1:1	Good separation
17	Hexane:ethyl acetate:formic acid	70:30:1	Good separation
18	Hexane:ethyl acetate:formic acid	70:20:1	Best separation

**Table 3:** Substance A–Q with their R_f_ and area percentage of *T. ammi* essential oil obtained by different extraction techniques.

Substance (R_f_)	Area Percentage (%) 254 nm				Area Percentage (%) 366 nm			
	HD	SE	US	SC-CO_2_	HD	SE	US	SC-CO_2_
A (0.01)	-	6.57	3.55	0.44	-	-	-	-
B (0.01)	-	-	-	-	-	14.93	11.36	-
C (0.09)	1.17	-	-	0.66	-	-	-	0.78
D (0.15)	-	-	-	-		-	-	3.01
E (0.2)	-	-	-	4.52	-	-	-	-
F (0.24)	-	1.74	-	-	-	20.90	18.16	-
G (0.3)	-	-	-	4.86	-	-	-	3.93
H (0.35)	-	1.88	2.00	-	-	18.96	21.78	4.45
I (0.37)	-	-	-	1.30	-	8.11	-	-
J (0.41)	-	-	1.35	-	-		3.25	-
K (0.44)	-	0.98	-	3.20	28.37	19.60	30.43	10.82
L (0.46)	3.50	-	5.09	-	-	11.14	15.02	-
M (0.58)	79.34	73.80	73.62	53.11	71.63	-	-	8.03
N (0.66)	8.49	5.19	-	-	-	6.36	-	68.99
O (0.71)	-	3.74	-	29.99	-	-	-	-
P (0.76)	5.31	6.10	14.39	-	-	-	-	-
Q (0.83)	2.20	-	-	1.91	-		-	-

R_f_: Retention factor, HD: Hydrodistillation, SE: Solvent extraction, US: Ultrasonication, SC-CO_2_: Supercritical carbondioxide extraction.

**Table 4:** Substance A–P with their R_f_ and area percentage of *T. ammi* essential oil obtained by different extraction techniques.

Substance (R_f_)	Area Percentage (%) 580 nm			
	HD	SE	US	SC-CO_2_
A (0.02)	-	0.59	2.21	-
B (0.06)	1.20	0.28	-	4.12
C (0.11)	-	0.41	-	1.66
D (0.16)	0.52	0.66	0.92	3.33
E (0.18)	0.82	-	-	-
F (0.25)	-	1.43	1.64	3.99
G (0.31)	-	9.29	-	7.98
H (0.33)	-	-	8.03	-
I (0.39)	18.80	6.42	4.88	-
J (0.44)	9.25	12.36	12.59	21.41
K (0.59)	61.94	27.17	30.34	28.94
L (0.72)	-	34.46	-	8.68
M (0.76)	-	-	30.23	12.75
N (0.78)	3.76	6.92	-	-
O (0.82)	4.52	-	9.14	7.02
P (0.92)	-	-	-	0.13

R_f_: Retention factor, HD: Hydrodistillation, SE: Solvent extraction, US: Ultrasonication, SC-CO_2_: Supercritical carbondioxide extraction.

**Table 5:** Results of the GC-MS analysis of *Tachyspermum ammi* oil extracted by different techniques.

Serial No	Component Name	Area Percent (%) 580 nm				
		RI	HD	SE	US	SC-CO_2_
Monoterpene Hydrocarbon						
1	3-Thujene	9052	1.20	0.51	-	-
2	(-)-β-Pinene	9110	-	-	2.31	0.62
3	l-Phellandrene	9745	-	-	-	0.41
4	α-Terpinene	9016	0.39	0.16	-	-
5	p-Cymene	9842	-	21.62	4.83	11.06
6	γ-Terpinene	9915	23.62	20.90	5.07	11.56
7	α-terpinolene	9004	0.15	-	-	-
8	Cis-sabinene hydrate	1195	-	-	-	0.21
Oxygenated Monoterpene						
9	Cymol	9816	32.78			
10	4-Thujanol	9732	-	-	0.15	0.21
11	Terpineol	9438	-		11.01	-
12	Thymol	9998	35.63	49.33	69.94	66.25
13	Carvacrol	9350	0.29	-	-	0.48
Sesquiterpene Hydrocarbons						
14	Trans-Caryophyllene	1462	-	-	-	0.09
15	β-Selinene	1499	0.02	0.07	-	0.11
16	α-selinene	1404	0.01	-	-	0.10
17	Eudesma-3,11-Diene	1417	-	0.06	-	-
18	Tumerone	1437	-	-	2.25	-
19	Curlone.α	1415	-	-	1.18	-
Oxygenated Sesquiterpenes Derivatives						
20	γ-Eudesmol	1468	-	-	-	0.29
21	Eudesm-4(14)-en-11-ol	1459	-	-	-	1.28
22	Dill-Apiol	1138	0.02	-	0.32	-
Aromatic compound						
23	Bicyclo[3.1.1]heptane, 6,6-dimethyl-2-methylene-, (1s)-	9056	3.54	-	-	-
24	Mentha-1,4,8-triene 1,5,8-p-menthatriene	9015	0.04	-	-	-
25	3-Cyclohexen-1-ol, 4-methyl-1-(1-methylethyl)	9980	0.50	-	0.15	0.25
	Non isoprenoid					
26	Linalyl propionate	1284	0.26	-	-	0.26
27	Heneicosane	2039	0.01	-	-	-
28	Pentadecane	1469	-	-	0.13	0.06
29	Phenol, 2,4-Bis (1,1-Dimethylethyl	1380	0.01	-	-	-
30	Dodecanoic acid, methyl ester	1276	-	-	-	0.09
31	Dodecane	1176	-	-	-	0.05
32	1,6,10-Dodecatrien-3-ol, 3,7,11-trimethyl-, [S-(Z)]-	1449	-	-	-	0.07
33	Hexadecane	1578	0.01	0.03	0.12	0.09
34	n-Heptadecane	1690	-	-	0.08	0.15
35	3 n -butyl Phthalide	1159	-	-	-	0.16
36	Octadecane	1785	0.01	0.03	0.27	-
37	Pentadecanoic acid, 14-methyl-, methyl ester	1665	-	-	0.85	-
38	Heneicosane	2098	-	-	0.42	0.10
39	Docosane	2145	-	0.11	-	0.07
40	9,12-Octadecadienoic acid (Z,Z)-, methyl ester	1896	-	0.39	4.27	0.53
41	Octadecanoic acid, methyl ester	1884	-	-	0.64	-
42	9-Octadecenoic acid	1786	-	0.83	-	-
43	Nonadecane	1890	0.03	0.05	0.06	0.17
44	Hexatriacontane	3597	-	-	0.78	0.14
45	Eicosane	1959	-	0.08	0.44	0.22
46	Pentacosane	2494	-	0.09	-	0.06
47	Tricosane	2389	-	-	0.07	0.28
48	Butyl pthalate	1575	0.18	-	0.91	0.37
49	Squalene	2997	-	0.08	0.17	-

RI: Retention indices, HD: Hydrodistillation, SE: Solvent extraction, US: Ultrasonication, SC-CO_2_: Supercritical carbondioxide extraction.

**Table 6:** Results of the FTIR analysis of *T. ammi* oil extracted by different techniques.

Serial No	Peak	Functional Group	Intensity	HD	SE	US	SC-CO_2_
1	810.1	=C-H(alkene)	stretch, strong	+	-	-	+
2	945.12	=C-H(alkene)	stretch, strong	+	-	-	+
3	1155.36	C-O(alcohol)	stretch, strong	+	+	-	+
4	1224.8	C-O( aldehyde)	stretch, medium	+	+	-	+
5	1284.59	C-O( aldehyde)	stretch, medium	-	+	-	+
6	1458.18	C=C(aromatic)	stretch, medium	+	+	-	+
7	1514.12	N-O(nitro)	stretch, strong	+	-	-	-
8	1616.35	N-O(nitro)	stretch, strong	+	-	-	+
9	2729.27	=C-H(aldehyde)	stretch, medium	+	+	-	+
10	2926.01	methylene	medium, strong	-	+	-	+
11	3007.02	C-H(aromatic)	stretch, medium	-	-	-	+
12	3429.43	N-H(amine)	stretch, medium	+	-	-	+

HD: Hydrodistillation, SE: Solvent extraction, US: Ultrasonication, SC-CO_2_: Supercritical carbondioxide extraction.

**Table 7:** Summary of % inhibition and IC_50_ values of the *T. ammi* oil sample by the DPPH method (*n* = 3).

Percentage Inhibition % (Mean ± SD7)		
Conc. (µg/mL)	Ascorbic Acid	*T. ammi*
100	92.5 ± 1.1	64.1 ±0.8
50	64.3 ± 0.9	56.7 ± 0.8
25	45.7 ± 1.0	41.5 ± 0.8
12.5	29.5 ± 0.54	34.1 ± 0.8
6.25	20.6 ± 0.8	28.9 ± 0.8
3.125	12.1 ± 0.8	20.9 ± 4.7
1.5625	8.45 ± 1.0	12.9 ± 0.8
0.781	5.85 ± 0.8	1.5 ± 0.8
IC_50_	28.09	36.41

SD: Standard deviation.

**Table 8:** Summary of % inhibition and *IC*_50_ values of the *T. ammi* oil sample by the superoxide anion scavenging method (*n* = 3).

Percentage Inhibition ± SD		
Conc. (µg/mL)	Ascorbic Acid	*T. ammi*
10	38.28 ± 0.8	39.18 ± 0.8
20	48.72 ± 1.2	43.88 ± 0.8
40	62.33 ± 0.8	57.69 ± 0.8
60	71.67 ± 1.2	77.03 ± 0.8
80	89.28 ± 1.2	85.76 ± 0.8
100	95.22 ± 1.2	93.00 ± 0.8
*IC*_50_	20.14	20.55

SD: Standard deviation.

**Table 9:** Estimated Effects and Coefficients for the Yield.

Term	Effect	Coef	SE Coef	T	P
Constant		1.09433	0.05154	21.23	0.000
Pressure	1.07339	0.53670	0.05318	10.09	0.000
Temperature	0.42795	0.21397	0.05414	3.95	0.000
Flow rate	0.67875	0.33937	0.05446	6.23	0.000
Pressure×Temperature	0.15887	0.07943	0.05431	1.46	0.155
Pressure×Flow rate	0.04000	0.02000	0.05446	0.37	0.716
Temperature×Flow rate	0.21875	0.10938	0.05446	2.01	0.054
Pressure×Temperature×Flow rate	−0.00250	−0.00125	0.05446	−0.02	0.982
